# Supplementary material for: Anti-tumor effects of P-LPK-CPT, a peptide-camptothecin conjugate, in colorectal cancer
Source: Commun Biol. 2022 Nov 14;5:1248. doi: 10.1038/s42003-022-04191-1 (PMC9663589; doi:10.1038/s42003-022-04191-1)
Supplement: Supplementary file 2 — Supplementary Information [file 42003_2022_4191_MOESM2_ESM.pdf]

Supplementary Methods

Cellular uptake of P-LPK-CPT

NCM460 and LoVo cells were seeded in glass bottom dishes at 1×10^5 per well. The cells were incubated with 100 nM free CPT, P-CON-CPT, P-LPK-CPT (with CPT equivalent dose of 100 nM) for 2 h. Then, culture medium was removed, and cells were washed with PBS for three times. Subsequently, the cells were fixed with 4 % paraformaldehyde for 30 min at room temperature, and the dishes were rinsed with PBS for 3 times. Finally, 100 μl (10 μg/ml) propidium iodide (PI) solution was added for 10 min. Images were taken from the PI channel (red), and the CPT channel (blue) by confocal microscope (CPT excitation: 370 nm, emission: 440 nm).

Transient gene transfection

Si-SLC1A5 and corresponding NC oligonucleotide sequences were synthesized by GenePharma (Shanghai, China). Transfection was performed with GP-transfect-Mate (GenePharma, Shanghai, China) at a final concentration of 200 nmol/L (siRNAs). Cells were harvested for assays 48 h after transfection.

Western blotting

Primary antibodies against SLC1A5 (Abcam, Cat No. ab237704,1:1000) and secondary antibodies labeled with HRP (YEASEN, 33701ES60, China,1:5000) was applied. Signals were detected using ECL Kit (Pierce Biotech, Rockford, IL). β-Actin (Sigma,USA, A5441) was performed as an internal control.

Supplementary figure legends

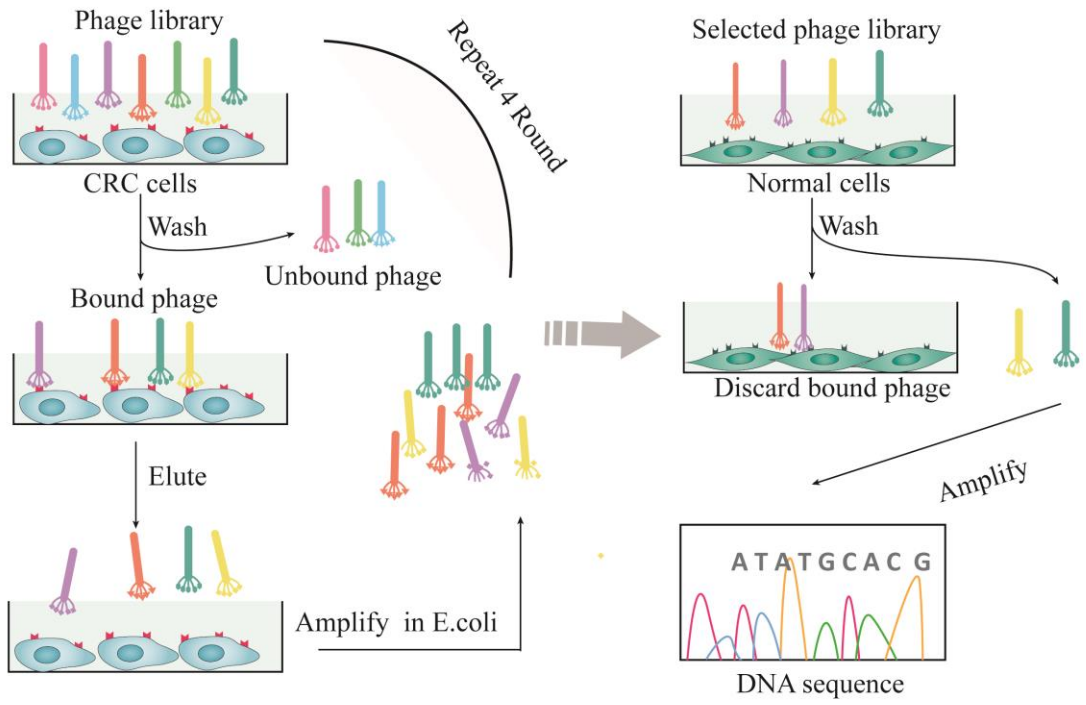

Supplementary Fig. 1 Schematic diagram of CRC specific binding peptide bio-panning process.

The phage random peptide library was first incubated with the CRC cells and the clones that did not bind to the CRC cells were washed away. Clones that bind to CRC cells were collected, enriched, and amplified. The amplified clones were incubated with CRC cells again. The screening process was repeated four times. Finally, the obtained phage clones are incubated with normal cells to remove non-specific clones, and ultimately obtain phage clones that specifically bind to CRC cells. The phage clones were sequenced to identify corresponding protein sequence.

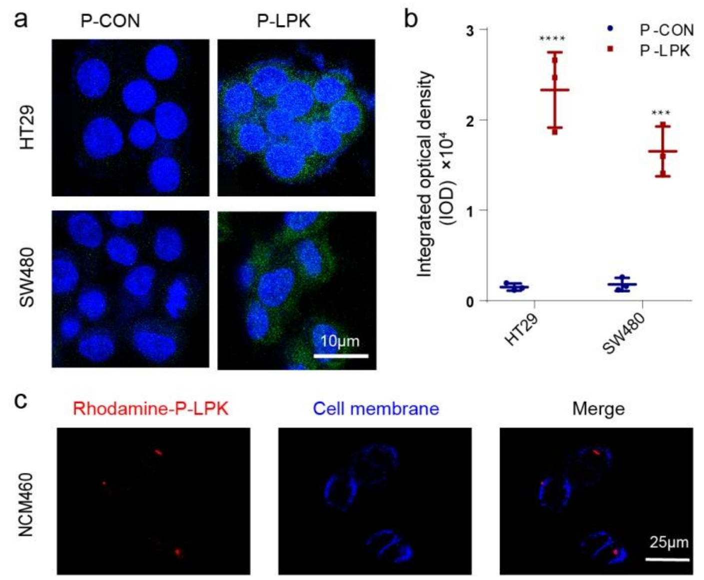

Supplementary Fig. 2 Fluorescent imaging of P-LPK binding to human CRC cells HT29 and SW480.

a 10μM FITC-P-LPK (Control group FITC-P-CON) were incubated with cells for 30 min and the binding ability to cells was observed by a confocal microscope. The results showed that P-LPK exhibited a stronger green fluorescence on HT29 and SW480, suggesting that P-LPK specifically binds to CRC cells. Bar, 10μm. **b** Fluorescence signal intensity analyzed by image pro plus software showed that the fluorescence intensity of P-LPK on CRC cells was significantly higher than that of P-CON (n = 3, means ± SD, HT29: P-CON vs P-LPK, p<0.0001; SW480: P-CON vs P-LPK, p=0.0002) (***p<0.001, ****p<0.0001). **c** The binding of the P-LPK peptide in human colonic epithelial cell NCM460 was investigated after the labeling of the peptide with Rhodamine. Bar, 25μm.

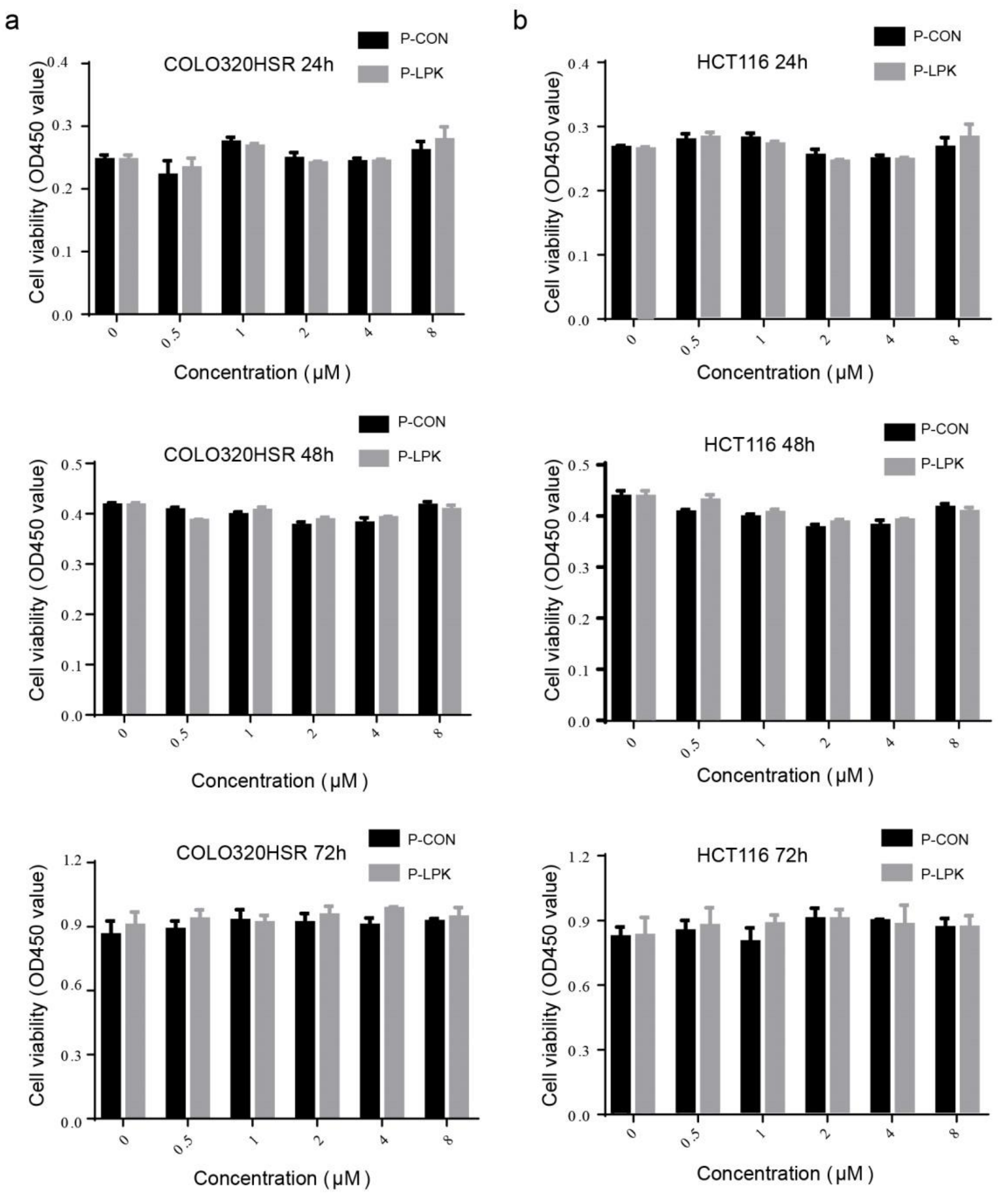

Supplementary Fig. 3 Effects of P-LPK on the proliferation of CRC cells.

a-b Different concentrations of P-LPK (Control peptide P-CON) were investigated in COLO320HSR (**a**) and HCT116 (**b**) for 24h, 48h, and 72h via CCK8 test. The results showed that P-LPK had no significant effects on proliferation of COLO320HSR cells and HCT116 cells at different concentrations and time points (n = 5, means ± SD) (P>0.05).

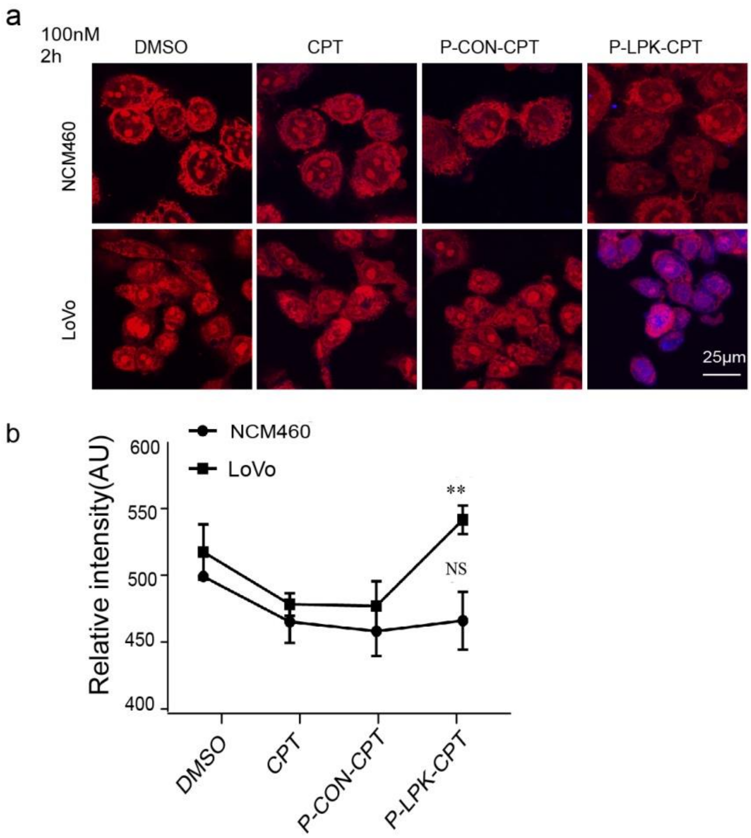

Supplementary Fig. 4 Intake of P-LPK-CPT of CRC cells.

a 100nM P-LPK-CPT (Control group DMSO, CPT, P-CON-CPT) was incubated with NCM460 or LoVo cells for 2h. The cellular intake of CPT was observed by confocal microscope. Images showed that P-LPK-CPT internalizes into LoVo cells but not NCM460 cells. Blue, CPT fluorescence. Red, PI (nucleic acid stain). **b** CPT fluorescence was detected by a fluorescence microplate reader. Compared with the control groups, the fluorescence intensity of P-LPK-CPT in LoVo cells increased significantly, while there was no significant difference in NCM460 in any groups (n = 3, means ± SD) (***P*< 0.01).

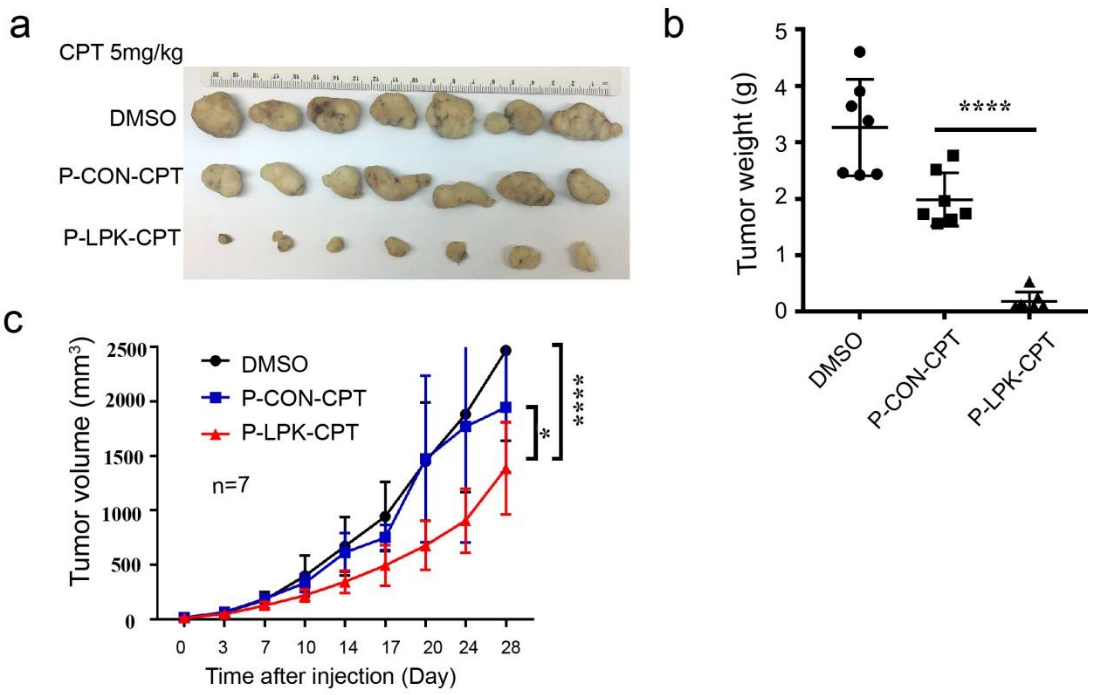

Supplementary Fig. 5 Treatment with P-LPK-CPT delays the growth of CRC xenografts *in vivo*.

The HCT116 tumor-bearing mice were randomly divided into four groups (n= 7 for each group). After the tumor size reached about 100 mm^3, the mice were intravenously injected DMSO, CPT, P-CON-CPT or P-LPK-CPT (equivalent CPT dose of 5.0 mg/kg) respectively, twice a week. More than half of the mice in the CPT group died during treatment, therefore CPT group was not shown in the figure. **a** Photographs of tumors after 28 days treatment in each group. **b** Tumor weights from different treatments. The results are presented as the arithmetic mean with standard deviation. There was a statistically significant difference between P-CON-CPT and P-LPK-CPT group (n = 7, means ± SD) (****P*< 0.001). **c** Tumor volume of the mice with different treatments within 28 days. The tumor volume growth rate of P-LPK-CPT group was remarkably decreased compared to control groups, suggesting that the antitumor effect of P-LPK-CPT was significantly enhanced. The results are presented as the arithmetic mean with standard deviation of tumor volumes in each group (n = 7, means ± SD) (**P*< 0.05, ****P*< 0.001).

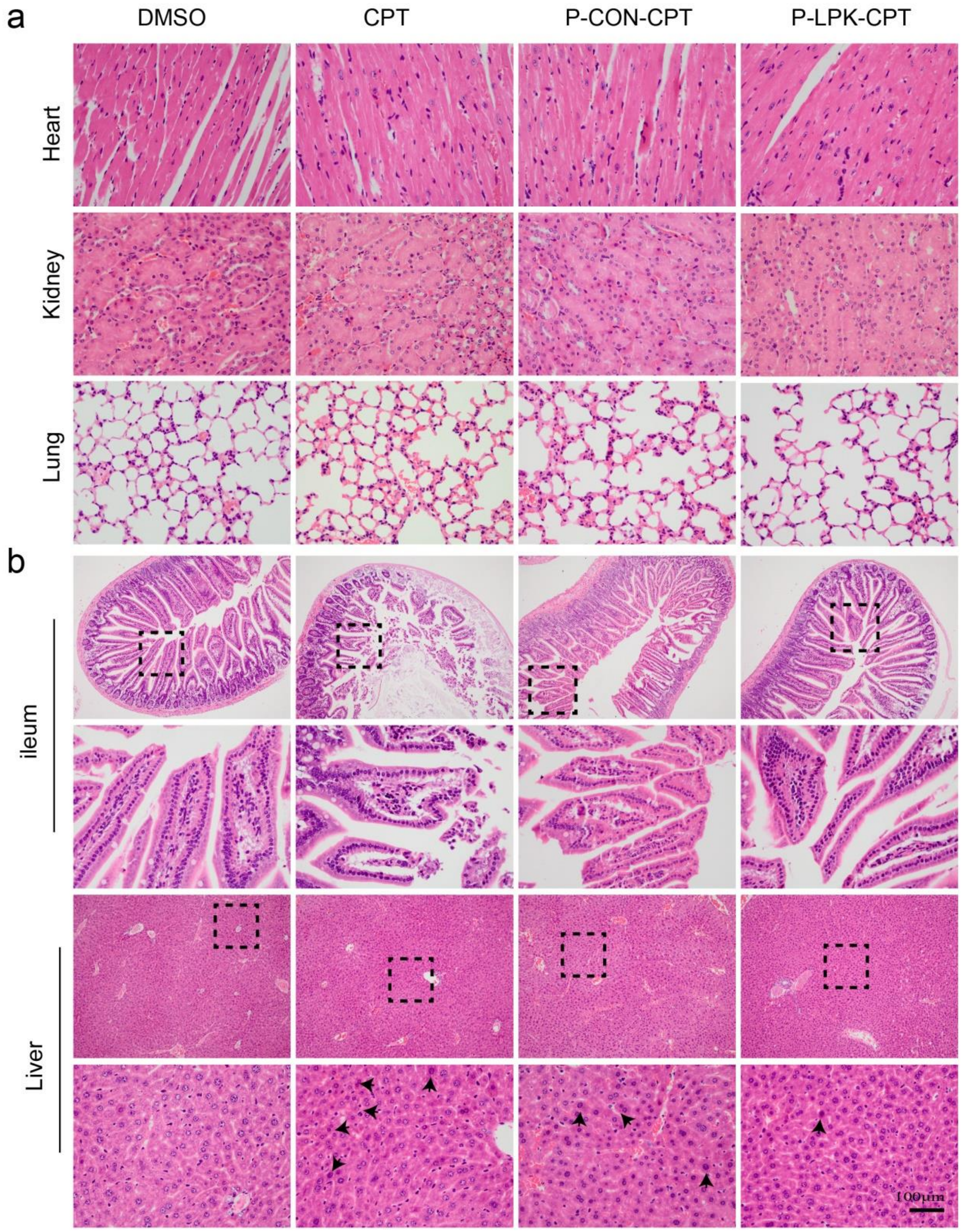

Supplementary Fig. 6 HE staining of different organ tissues in PDX model mice.

At the end of treatments, nude mice were sacrificed. HE staining was used to observe the effect of different treatments on vital organs (heart, kidneys, lung, ileum, liver).

a In the four groups, no obvious inflammation and necrosis were observed in heart, kidney, lung. Moreover, barely significant mutative morphology and structures were found in these organs. **b** The villi of the intestinal mucosa were destroyed and the structure of intestinal epithelial barrier was incomplete in CPT group, suggesting that

the damage of CPT to intestinal epithelium was significantly increased compared with. Meanwhile, apoptotic hepatocytes (arrow) are mostly obvious in the CPT group compared with the other groups. These findings showed P-LPK-CPT can effectively reduce the damage of CPT to intestinal and liver.

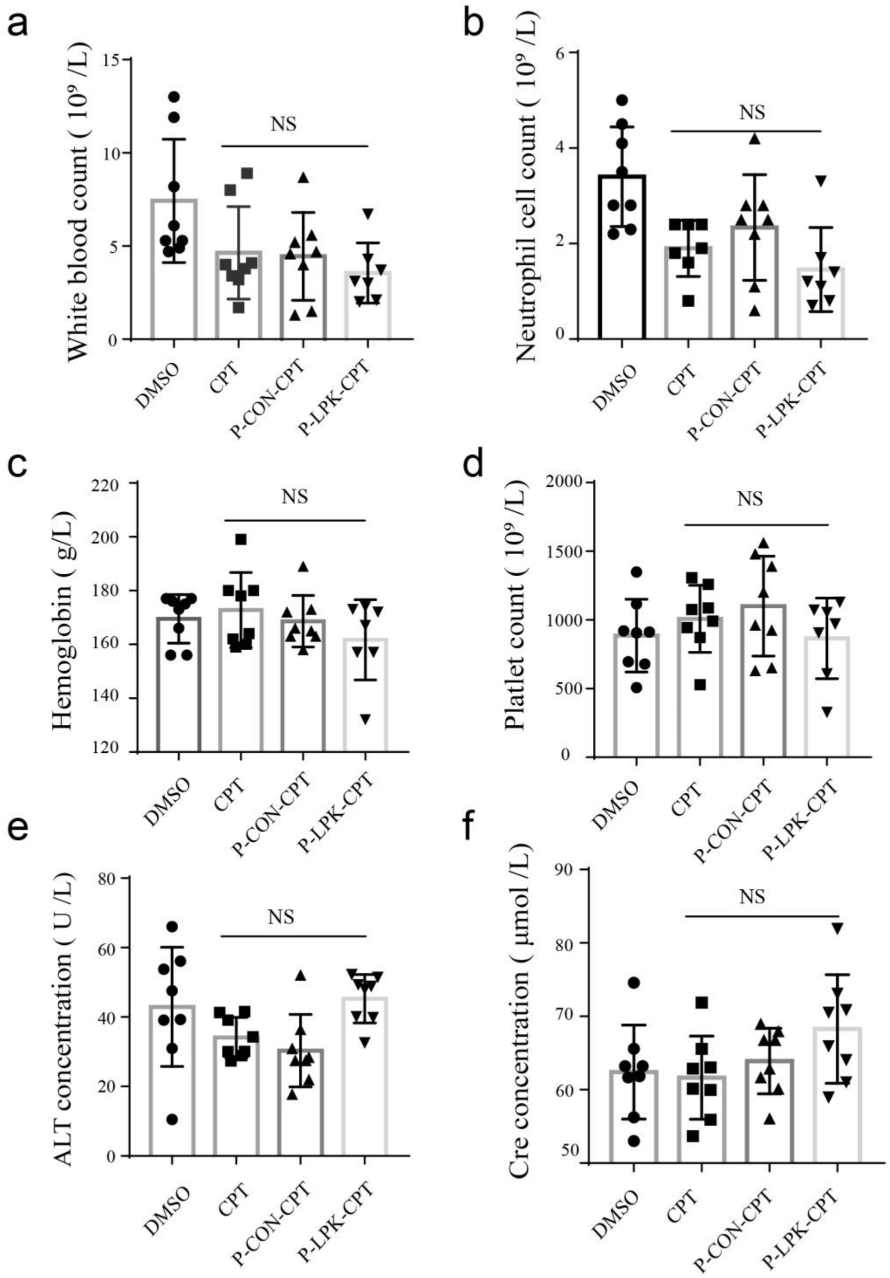

Supplementary Fig. 7 Routine blood cell test and biochemistry assays in mice following different treatments.

a-d Blood cells collected in EDTA capsule tubes were analyzed by WBC, Neutrophil, Hb and platelet count. The other part of blood samples was centrifuged, and the plasma was used for biochemistry assays (alanine aminotransferase (ALT) and creatinine (Cre). These tests were executed by routine clinical laboratory tests.

Treatment with P-LPK-CPT has no significant effect on blood cell counts ($P > 0.05$). **e-f** The effects of P-LPK-CPT on liver and kidney were detected by the biochemistry assays. There is no marked elevation of the ALT (**e**) and Cre (**f**) concentration after P-LPK-CPT treatment, and no clinical signs of toxicity were observed in liver and kidney. Values are presented as means ± SD .

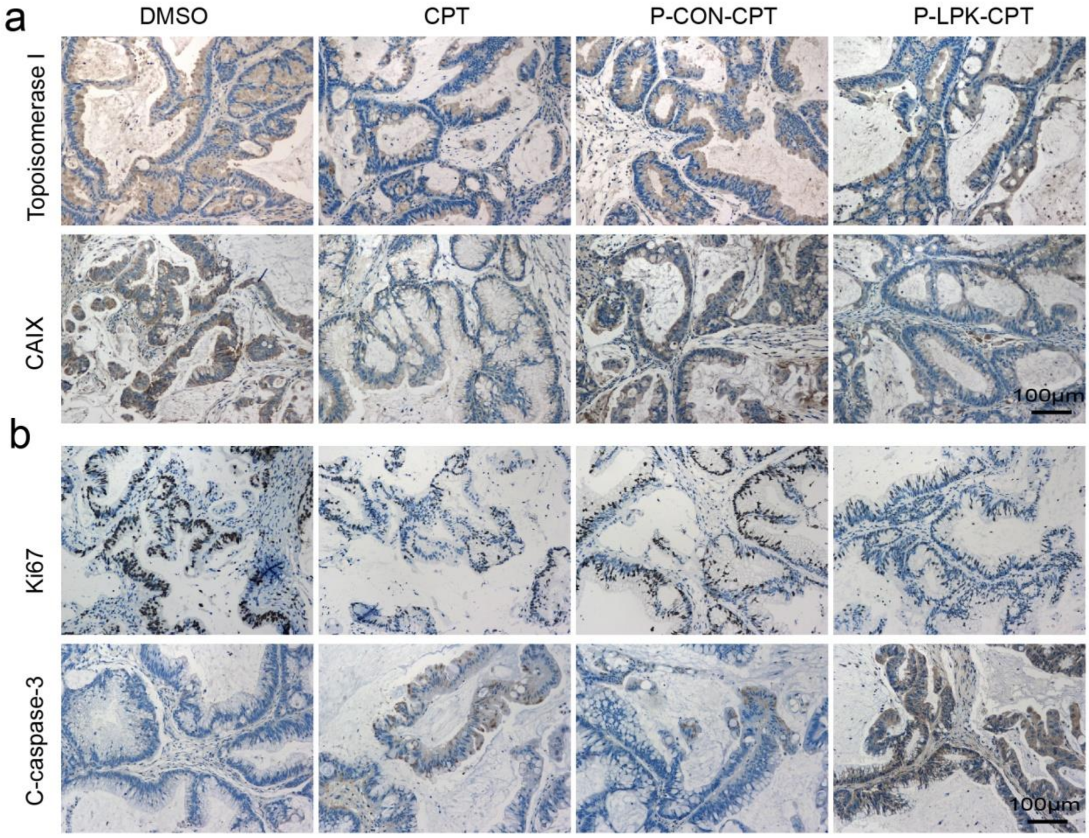

Supplementary Fig. 8 Immunohistochemistrty assays of CPT-related proteins and proliferation-related proteins.

a IHC staining on tumor sections harvested after treatment with different groups showed that P-LPK-CPT was able to inhibit both topoisomerase I (TOPOI) and downstream carbonic anhydrase IX (CAIX) expression compared with control groups (DMSO, CPT, P-CON-CPT). **b** In the Ki67 and C-casepase3 staining, scattered brown positive staining was observed in the tumor tissues. Ki67 expression in tumor tissue of P-LPK-CPT group was significantly lower than that of control groups but the expression of C-casepase3 increased significantly, suggesting that P-LPK-CPT inhibit cancer cell proliferation, as well as promote cell apoptosis in tumor tissues.

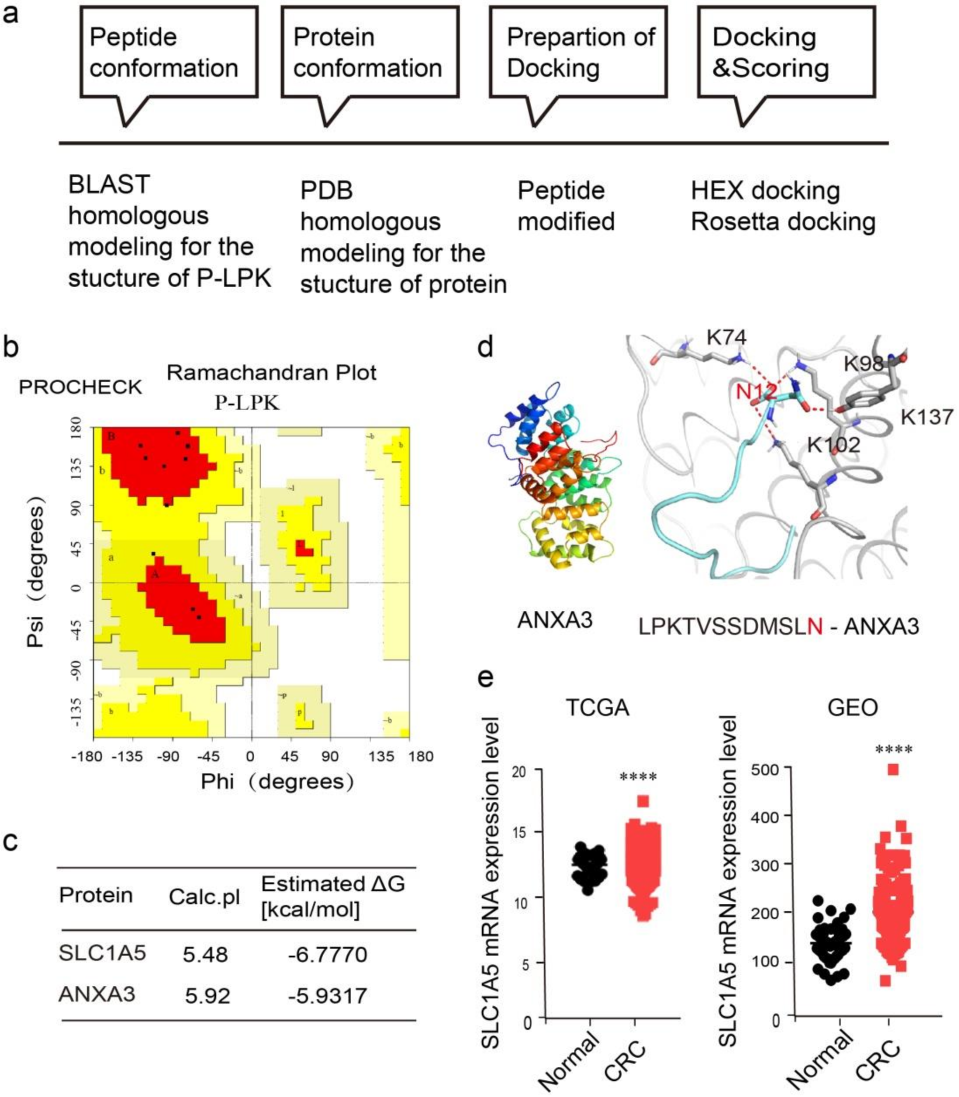

Supplementary Fig. 9 SLC1A5 was identified as the most possible candidate receptor of P-LPK.

a Flow diagram of molecular docking between P-LPK and candidate receptors. **b** Ramachandran plot of P-LPK. **c** The possible minimum-energy conformations of the peptide binding sites of SLC1A5 and ANXA3 were probed using RosettaDock with Monte Carlo-based algorithm. **d** Molecular interactions of P-LPK to ANXA3 were predicted by molecular docking. **e** SLC1A5 expression was analyzed from CRC TCGA and GEO database (accession number is GSE41258) (*****P*< 0.0001).

SLC1A5 siRNA oligo

si-NC	GCGACGAUCUGCCUAAGAUdTdT
si-SLC1A5（1230)	TCGCTCATACTCTACCACCTA
si-SLC1A5（1234)	mGmCmTCATACTCTACCACmCmTmAdTdT
si-SLC1A5（1529)	mCmCmGCCTTGGCAAGTACmAmTmTdTdT
si-SLC1A5（2017)	mGmCmACAGAGCCTGAGTTmGmAmTdTdT

Supplementary Fig. 10 4 siRNA sequence for SLC1A5 and negative control-siRNA.

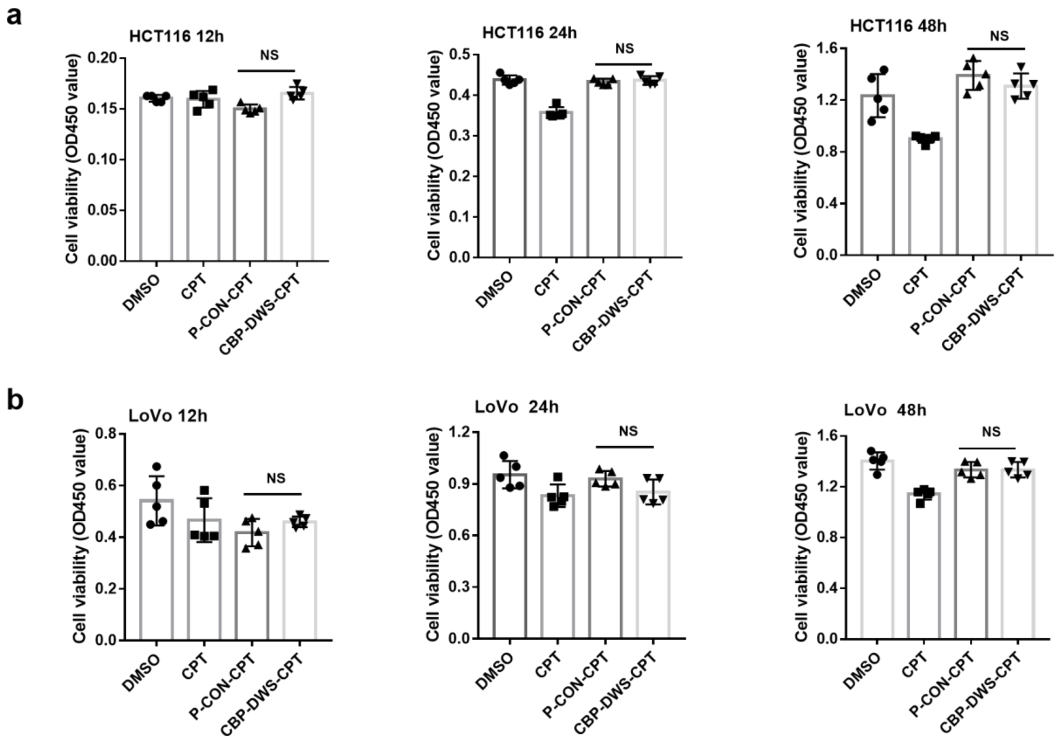

Supplementary Fig. 11 *In vitro* cytotoxicity of the CBP-DWS-CPT conjugate

a-b The CBP-DWS-CPT conjugate (Control group DMSO, CPT, P-CON-CPT) was incubated with CRC cells for 12h, 24h, and 48h. Cell proliferation activity was detected by CCK-8. The CBP-DWS-CPT conjugate exhibited no significant anti-tumor effect on HCT116 (**a**) and LoVo cells (**b**) (n = 5, means ± SD) (p>0.05).

HCT116 12h

DMSO	CPT	P-CON-CPT	CBP-DWS-CPT
0.163	0.162	0.146	0.163
0.157	0.155	0.149	0.163
0.157	0.169	0.157	0.168
0.163	0.164	0.148	0.175
0.163	0.148	0.151	0.159

HCT116 24h

DMSO	CPT	P-CON-CPT	CBP-DWS-CPT
0.426	0.352	0.442	0.433
0.438	0.355	0.426	0.428
0.439	0.381	0.432	0.446
0.456	0.351	0.426	0.45
0.431	0.349	0.441	0.427

HCT116 48h

DMSO	CPT	P-CON-CPT	CBP-DWS-CPT
1.211	0.921	1.247	1.324
1.436	0.849	1.403	1.203
1.369	0.903	1.526	1.326
1.127	0.923	1.466	1.456
1.034	0.911	1.317	1.237

LoVo 12h

DMSO	CPT	P-CON-CPT	CBP-DWS-CPT
0.45	0.405	0.358	0.458
0.462	0.409	0.371	0.444
0.519	0.404	0.476	0.436
0.674	0.582	0.463	0.487
0.601	0.532	0.423	0.473

LoVo 24h

DMSO	CPT	P-CON-CPT	CBP-DWS-CPT
1.001	0.826	0.886	0.786
1.064	0.798	0.906	0.806
0.888	0.823	0.97	0.809
0.878	0.941	0.986	0.932

0.936	0.768	0.899	0.931

LoVo 48h

DMSO	CPT	P-CON-CPT	CBP-DWS-CPT
1.398	1.144	1.395	1.395
1.482	1.179	1.265	1.265
1.431	1.159	1.294	1.294
1.411	1.068	1.4	1.4
1.296	1.161	1.317	1.317

Supplementary Fig.12 Uncropped and unedited blot images behind Fig. 6a

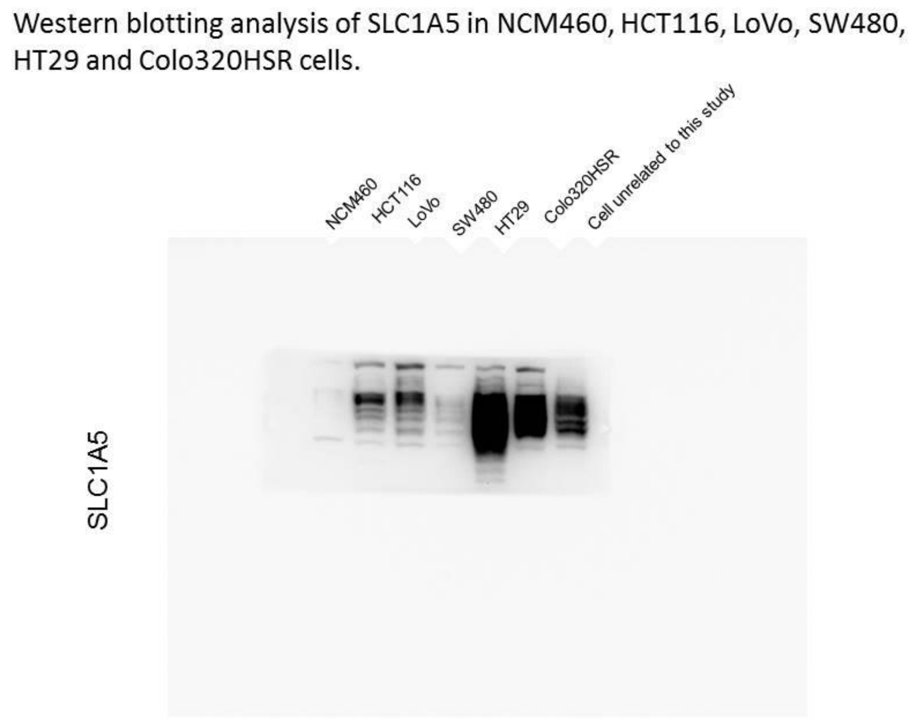

Western blotting analysis of SLC1A5 in NCM460, HCT116, LoVo, SW480, HT29 and Colo320HSR cells.

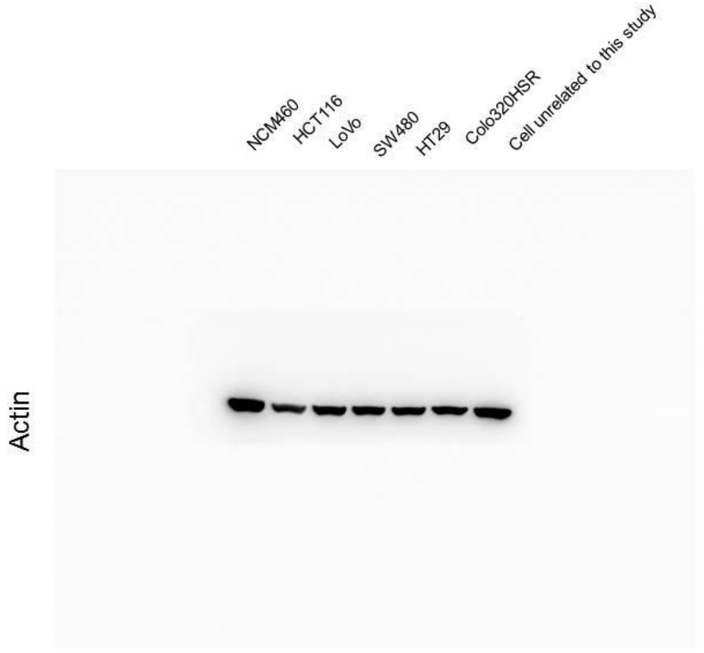

Knock down SLC1A5 in HCT116

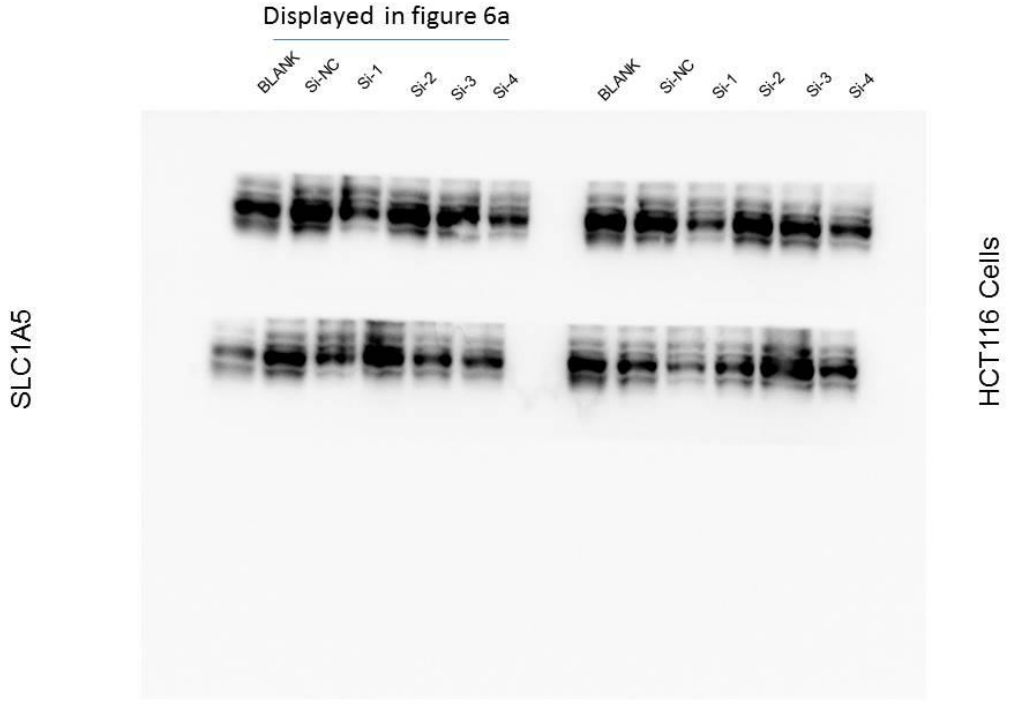

Knock down SLC1A5 in HCT116

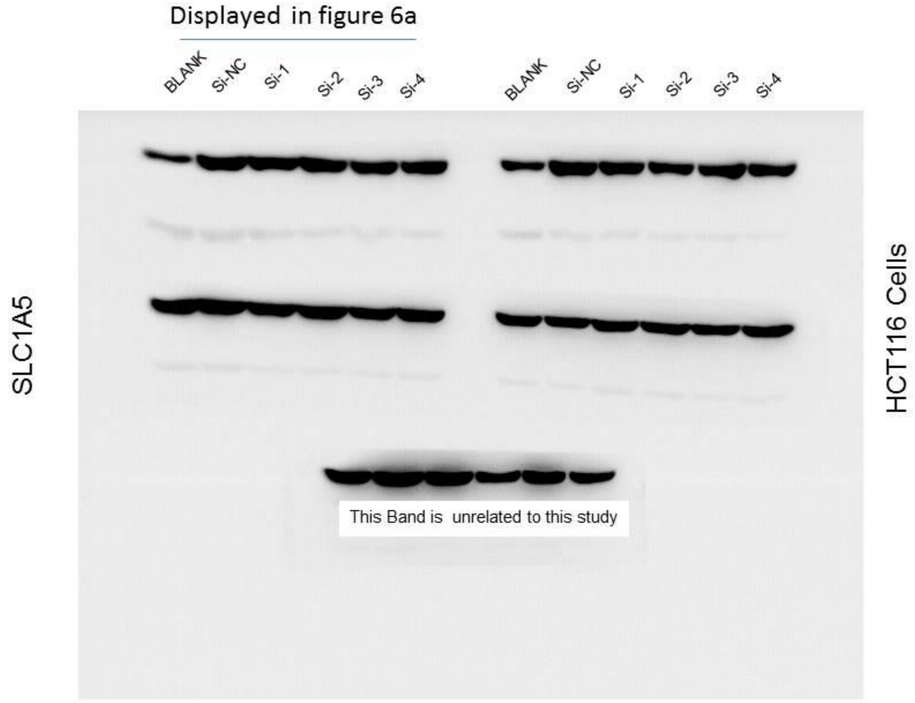

Knock down SLC1A5 in HT29

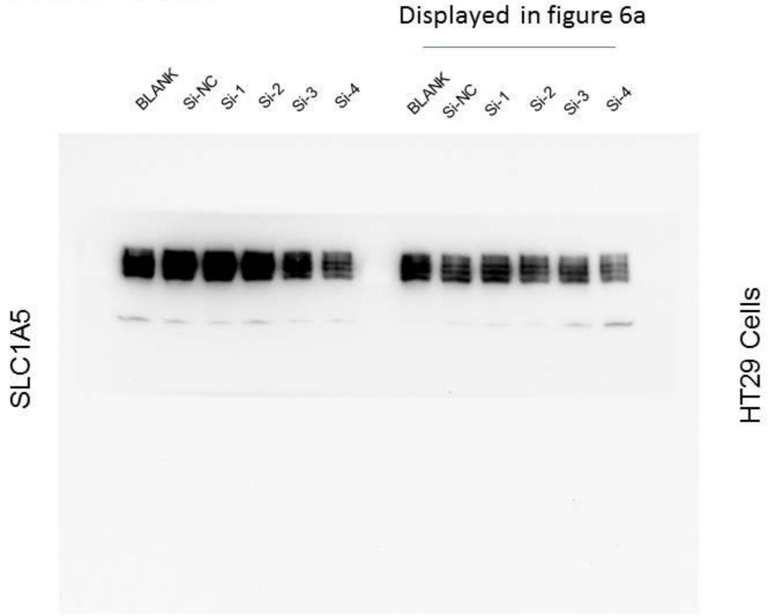

Knock down SLC1A5 in HT29

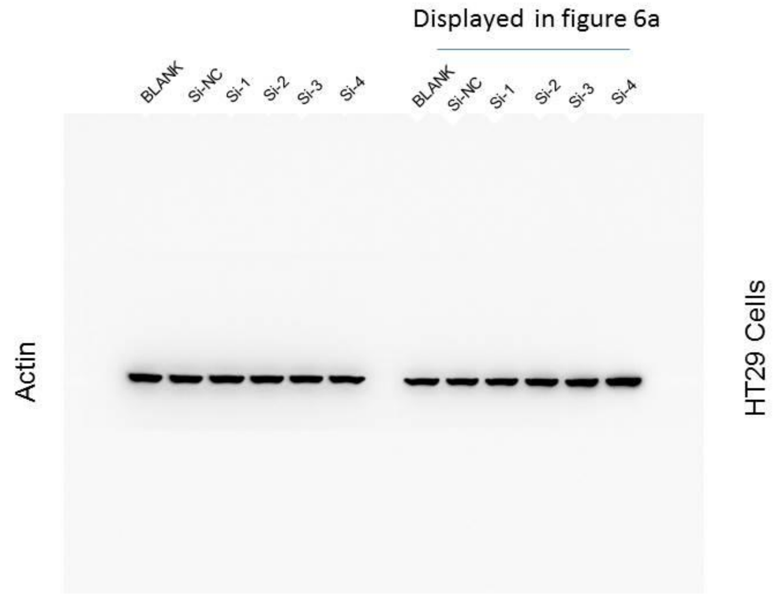

Supplementary Table 1 Validation of the selected peptide P-LPK through the analysis of existing target-unrelated peptides (TUPs), false positives and/or mimotopes.

Analysis	P-LPK: LPKTVSSDMSLN
TUPscan for target-unrelated peptides	No motif encountered
Mimoscan for published mimotopes of other researchers with various target peptides	No hits found, indicating that the peptide is target specific
Mimoblast to check mimotope database for peptide identity	E-value* too high, indicating non-reliable similarity to existing peptides

SAROTUP is freely available at http://i.uestc.edu.cn/sarotup3

Supplementary Table2 Homology analysis of P-LPK using protein-prptein blast BLASTP 2.6.0+

Homologous protein	Query Range	Identities	Gaps	E-value	Accession Number
Immunoglobulin heavy chain partial	4 T-S- DMSLN12<br>68 TISVDMSLN76	7/9	0/9	10	AAT38468.1
Cell division cycle associated 4	3 KTVSS---DMSL11<br>25 KTVSS---DMSL 42	9/18	9/18	21	AAH25263.1
hCG1645559, partial	2 PKT—VSSDMS 10<br>220 PKTLDVDSDMS 230	8/11	2/11	42	EAW77018.1
family with sequence similarity 70, member A, isoform CRA_a	6 SSDMSL 11<br>10 SSDMSL 15	6/6	0/6	60	EAX11887.1
coiled-coil-helix-coiled-coil-helix domain containing 3, isoform CRA_a	6 SSDMSL 11<br>37 SSDMSL 42	6/6	0/6	60	EAW83800.1
glutaredoxin-like protein C5orf63 isoform 1	2 PKT-SSD 8<br>109 PKTISSD 115	6/7	0/7	60	NP_001157951.1
transmembrane protein 255A isoform 3	6 SSDMSL 11<br>10 SSDMSL 15	6/6	0/6	60	NP_001157951.1
E3 ubiquitin-protein ligase TRIM7 isoform 2	2 P-TSSDM 9<br>118 PTTVSSEM 125	6/8	0/8	60	NP_976042.1
MHC class I antigen	6 SSDMSL 11<br>355 SSDMSL 360	6/6	0/6	60	SMB18740.1
KIAA0518 protein, partial	1 LPK—S-DM 9<br>328 LPKKISGDM 336	6/9	0/9	60	BAA25444.1
