# Supplementary material for: Anti-tumor effects of P-LPK-CPT, a peptide-camptothecin conjugate, in colorectal cancer
Source: Commun Biol. 2022 Nov 14;5:1248. doi: 10.1038/s42003-022-04191-1 (PMC9663589; doi:10.1038/s42003-022-04191-1)
Supplement: Supplementary file 3 — Description of Additional Supplementary Files [file 42003_2022_4191_MOESM3_ESM.pdf]

Description of Additional Supplementary Files

File name: Supplementary Data 1

Description: 241 proteins relatively specific to P-LPK were identified.

File name: Supplementary Data 2

Description: The most possible binding partner for the P-LPK peptide was analyzed

File name: Supplementary Data 3

Description: Source data underlying the graphs in excel format
